# Supplementary material for: Light-responsive MXenegel via interfacial host-guest supramolecular bridging
Source: Nat Commun. 2024 Jan 31;15:916. doi: 10.1038/s41467-024-45188-0 (PMC10831044; doi:10.1038/s41467-024-45188-0)
Supplement: Supplementary file 1 — Supplementary Information [file 41467_2024_45188_MOESM1_ESM.pdf]

Supplementary Information

Light-Responsive MXenegel via Interfacial Host-Guest Supramolecular Bridging

Yu-Liang Lin,[1] Sheng Zheng,[1] Chun-Chi Chang,[1] Lin-Ruei Lee,[1] and Jiun-Tai Chen[1,2,*]

[1]Department of Applied Chemistry, National Yang Ming Chiao Tung University, Hsinchu 300093, Taiwan

[2]Center for Emergent Functional Matter Science, National Yang Ming Chiao Tung University, Hsinchu 300093, Taiwan

[*]To whom correspondence should be addressed. Email: jtchen@nycu.edu.tw. Tel: 886-3-5731631

Synthesis of 4-(phenyldiazenyl)phenol (AzoOH)

First, 5g (72.47 mmol) $NaNO_2$ was dissolved in 50 mL of deionized water and added to a solution of 5.27 g aniline (56.62 mmol) and 50 mL of deionized water at 4 °C. Then, 17 mL of concentrated HCl was added slowly into the mixture. To prevent the reaction from overheating, a small amount of crushed ice (10 g) was also added. The mixture was stirred in an ice bath for 10 min to form a diazonium salt solution. Next, a solution of sodium hydroxide (2 g, 50.0 mmol), sodium carbonate (4.80 g, 45.29 mmol), and phenol (5.33 g, 56.6 mmol) in 50 ml deionized water was slowly added. The reaction mixture was stirred for 1 h at 0 °C, and then the reaction temperature was raised to room temperature. After the reaction, the mixture was acidified with concentrated HCl. The yellow precipitate was filtered and washed several times with cold deionized water. Purification was performed by reprecipitating with the mixture of MeOH and water (1:1, v/v). Finally, the product was dried in a vacuum oven for 24 h at 60 °C. Yield: 90%. ^{1}H NMR (400 MHz, Chloroform-*D*) δ 7.91−7.84 (m, 4H), 7.54−7.40 (m, 3H), 6.98−6.92 (m, 2H), 5.34 (s, 1H).

Synthesis of 1-(4-((6-bromohexyl)oxy)phenyl)-2-phenyldiazene (AzoBr)

2.0 g (10.09 mmol) 4-(phenyldiazenyl)phenol (AzoOH) was dissolved in 25 mL acetonitrile. Then, 2.95 g (12.11 mmol) 1,6-dibromohexane and 1.38 g (10 mmol) potassium carbonate were added to the solution. The reaction was carried out at 80 °C in an oil bath for 24 h. The reaction was cooled down to room temperature, and 250 mL of deionized water was added. The precipitate was filtered and washed with cold deionized water. Subsequently, the product was recrystallized with 2-propanol and dried in a vacuum oven. Yield: 92%.^{1}H NMR (400 MHz, Chloroform-*D*) δ 8.04−7.81 (m, 4H), 7.54−7.40 (m, 3H), 7.02−6.98 (m, 2H), 4.05 (t, *J* = 6.4 Hz, 2H), 3.44 (t, *J* = 6.8 Hz, 2H), 1.87 (dt, *J* = 20.4, 6.8 Hz, 2H).

Synthesis of 1-methyl-3-(5-(4-(phenyldiazenyl)phenoxy)pentyl)-1H-imidazol-3-ium (AzoC6)

First, in a round-bottom flask, 2 g (6.55 mmol) AzoBr and 0.64 g (7.86 mmol) 1-methylimidazole, 1 g (7.23 mmol) potassium carbonate, and 50 mL of acetonitrile were added and heated to 80 °C to fully dissolve the reagents. Then, the mixture was refluxed for 24 h at 120 °C under a nitrogen atmosphere. After cooled down to room temperature, the mixture was precipitated by diethyl ether. The precipitate was dissolved by ethanol and then precipitated by diethyl ether. The dissolution-precipitation process was repeated three times. The product was dissolved in the mixture of isopropyl alcohol and ethyl acetate (1:1, v/v), and the precipitate was filtered. The supernatant was dried by rotary evaporation using a vacuum pump. Yield: 63%.^{1}H NMR (400 MHz, Chloroform-*D*) δ 10.76−10.67 (m, 1H), 7.94−7.84 (m, 4H), 7.50 (t, J = 7.5 Hz, 2H), 7.43 (t, J = 7.2 Hz, 1H), 7.27−7.21 (m, 2H), 6.99 (d, J = 8.9 Hz, 2H), 4.36 (t, J = 7.5 Hz, 2H), 4.10 (s, 3H), 4.04 (t, J = 6.3 Hz, 2H), 1.98 (p, J = 7.6 Hz, 2H), 1.83 (p, J = 6.8 Hz, 2H), 1.57 (p, J = 7.4 Hz, 2H), 1.46 (p, J = 7.6 Hz, 2H); ^{13}C NMR (125 MHz, Chloroform-*D*) δ 161.62, 152.89, 147.07, 138.65, 130.53, 129.19, 124.91, 123.93, 122.69, 119.91, 114.84, 67.98, 50.26, 36.90, 30.33, 28.97, 26.03, 25.59; HR-ESI (m/s): [M]+ calcd. for C22H27N4O, 363.2179; found, 363.2181.

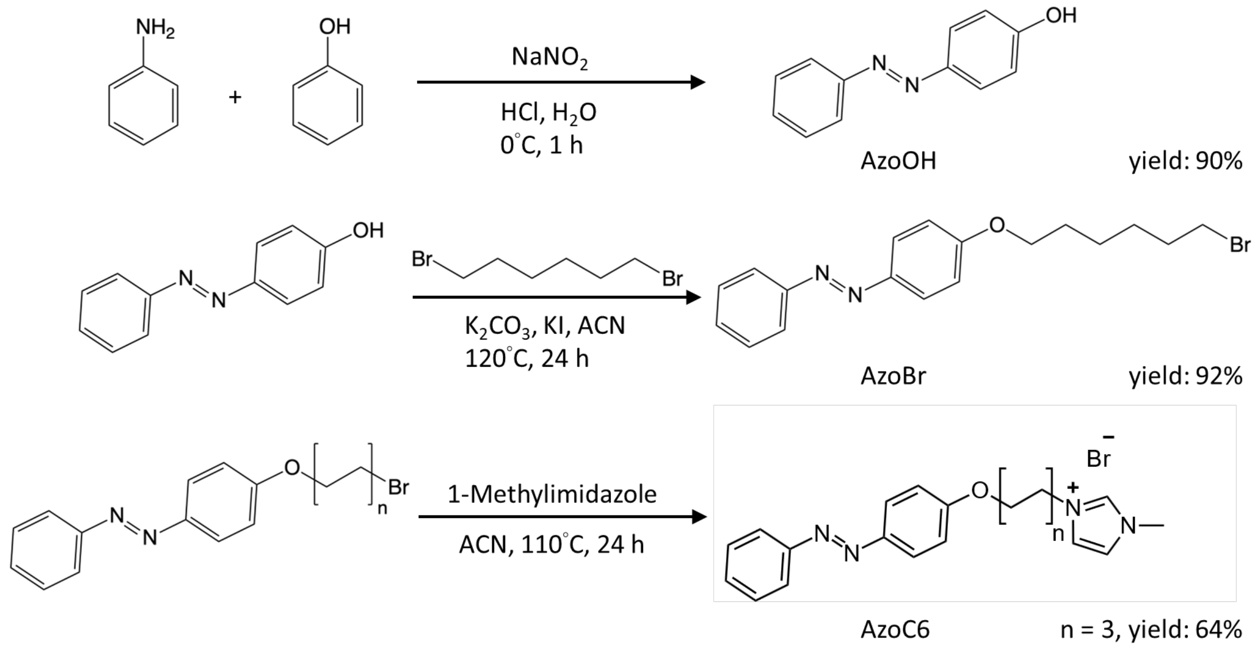

Supplementary Fig. 1. Synthetic scheme of AzoC6.

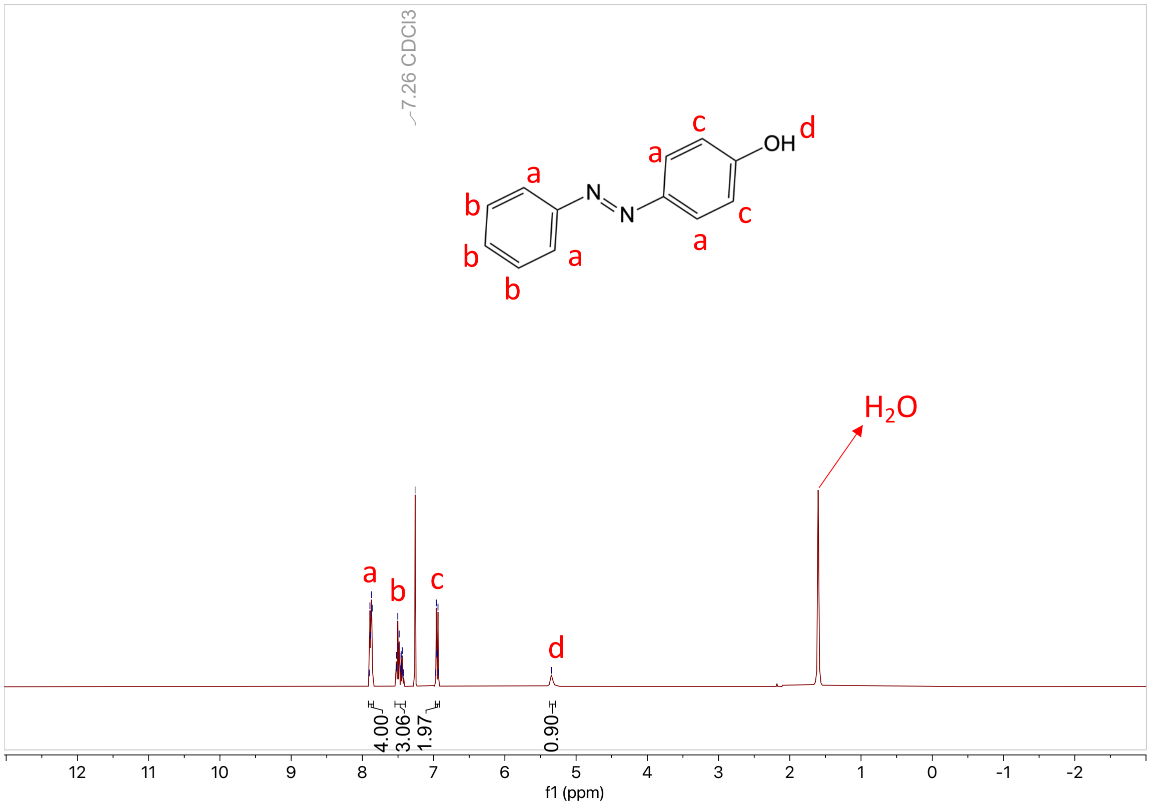

Supplementary Fig. 2. ^{1}H NMR spectrum (400 MHz, $CDCl_3$, 298 K) of AzoOH.The characteristic peaks are indicated.

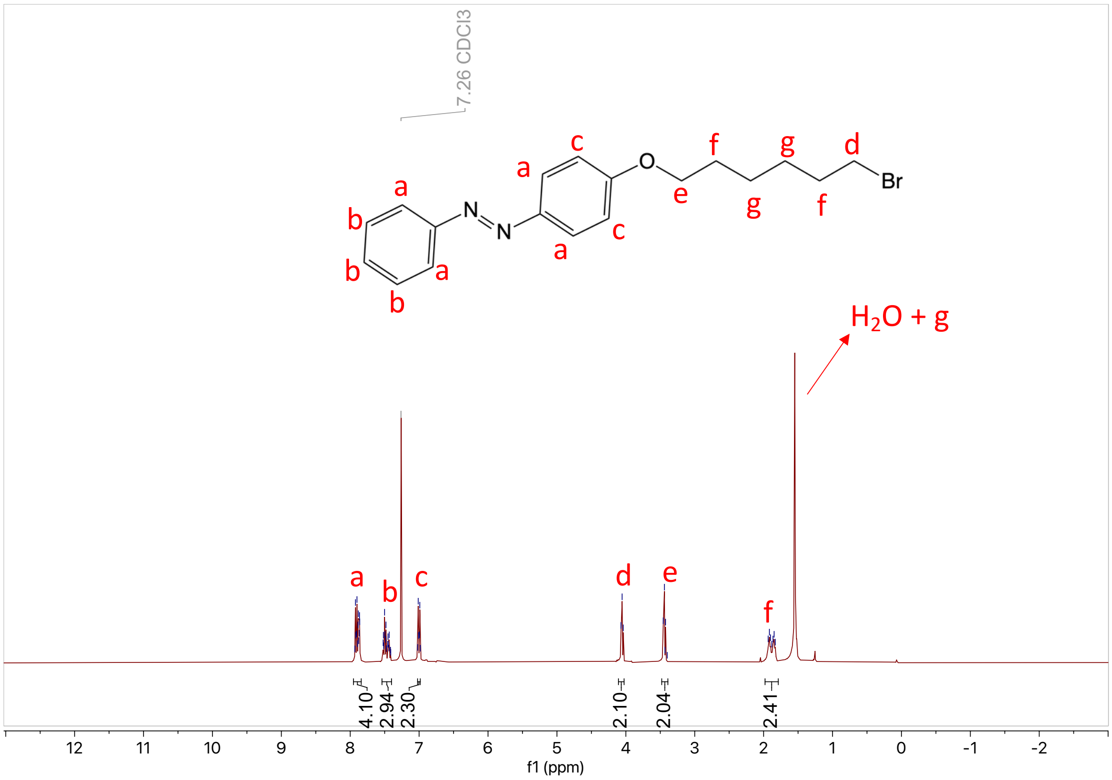

Supplementary Fig. 3. ^{1}H NMR (400 MHz, $CDCl_3$, 298 K) spectrum of AzoBr. The characteristic peaks are indicated.

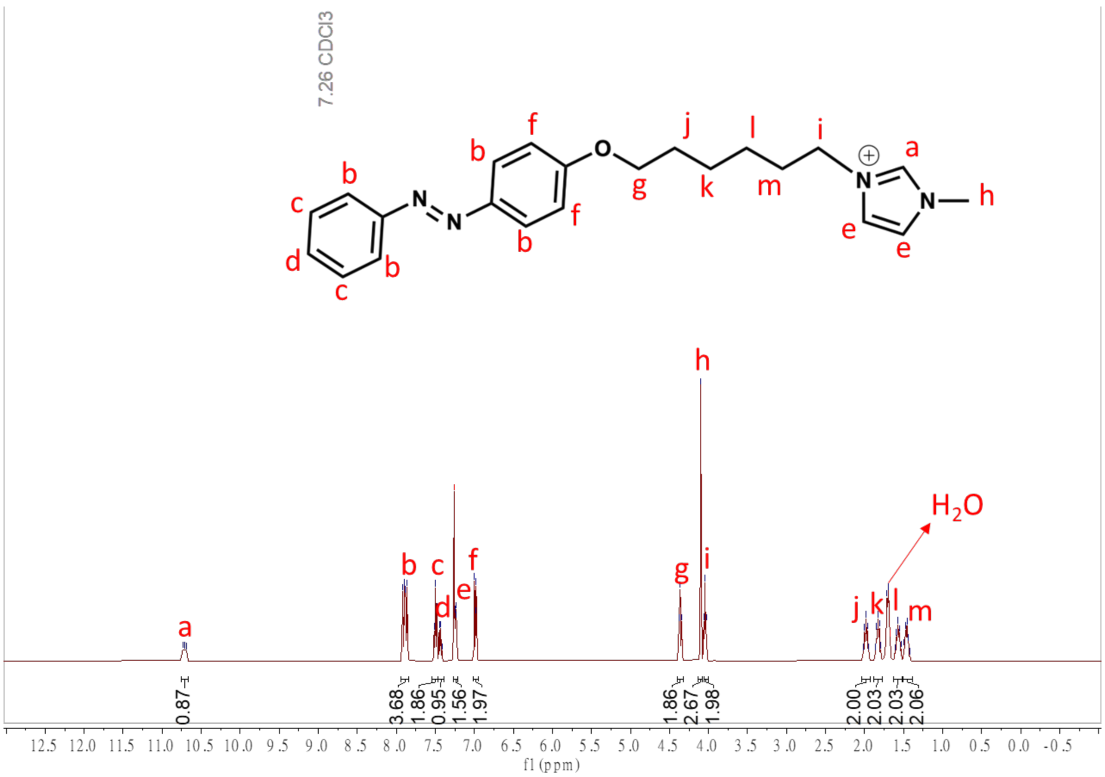

Supplementary Fig. 4. ^{1}H NMR spectrum (400 MHz, $CDCl_3$, 298 K) of AzoC6. The characteristic peaks are indicated.

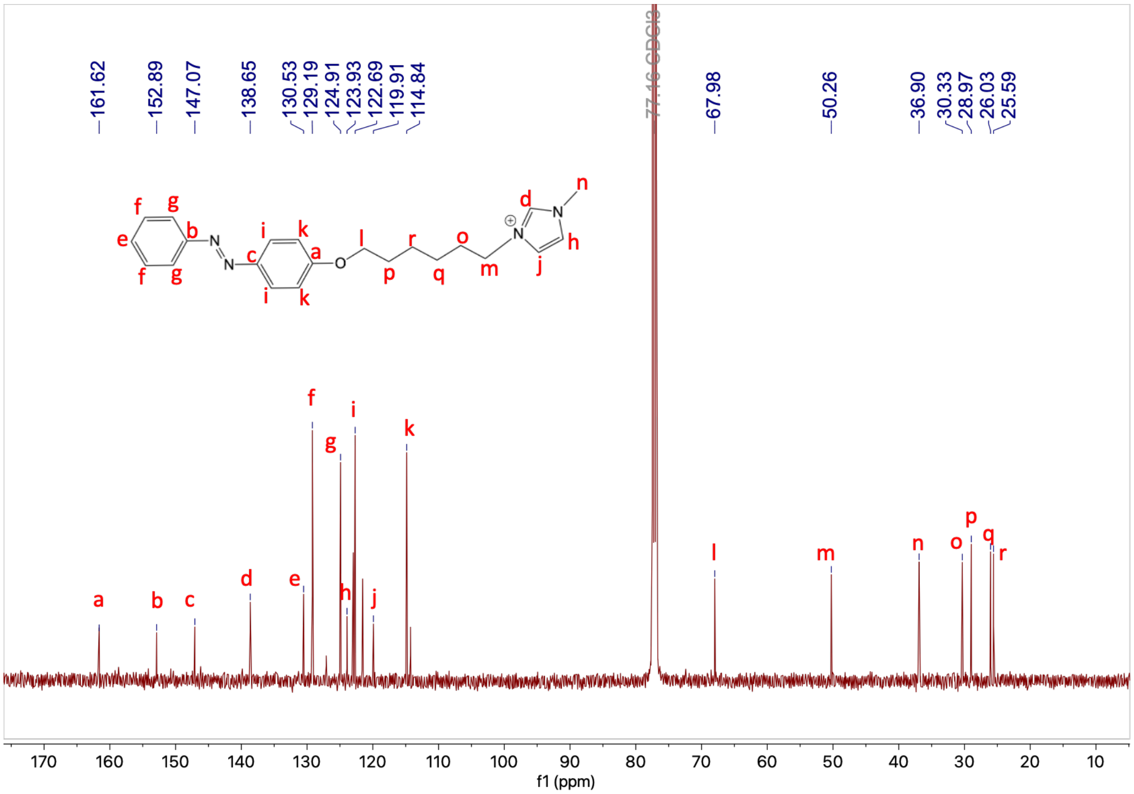

Supplementary Fig. 5. ^{13}C NMR spectrum (125 MHz, $CDCl_3$, 298 K) of AzoC6. The characteristic peaks are indicated.

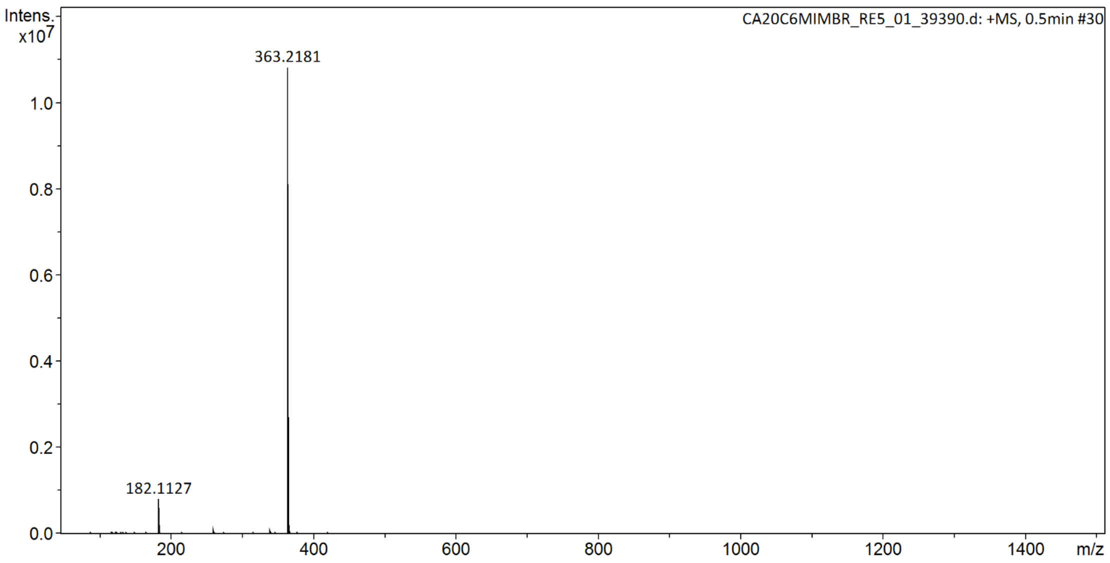

Supplementary Fig. 6. HR-ESI mass spectrum AzoC6.

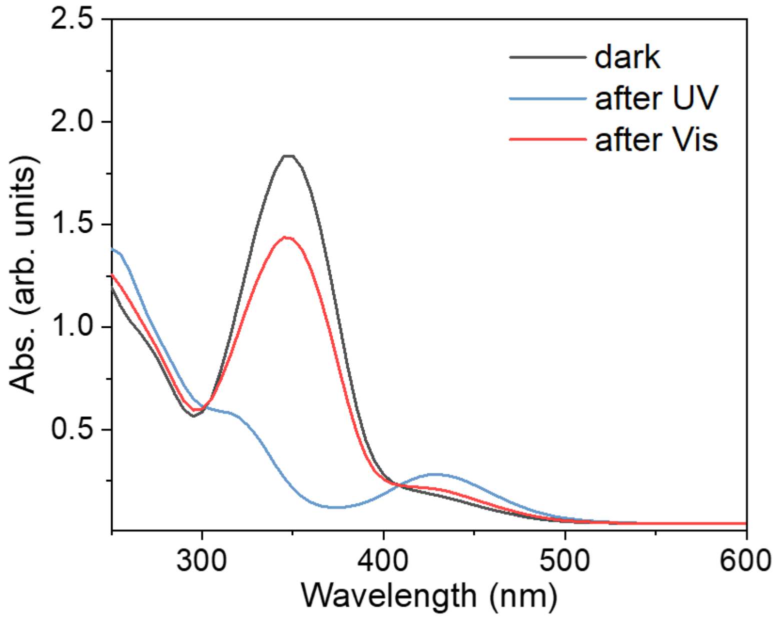

Supplementary Fig. 7. UV−vis spectra of AzoC6 in water under different light irradiations.

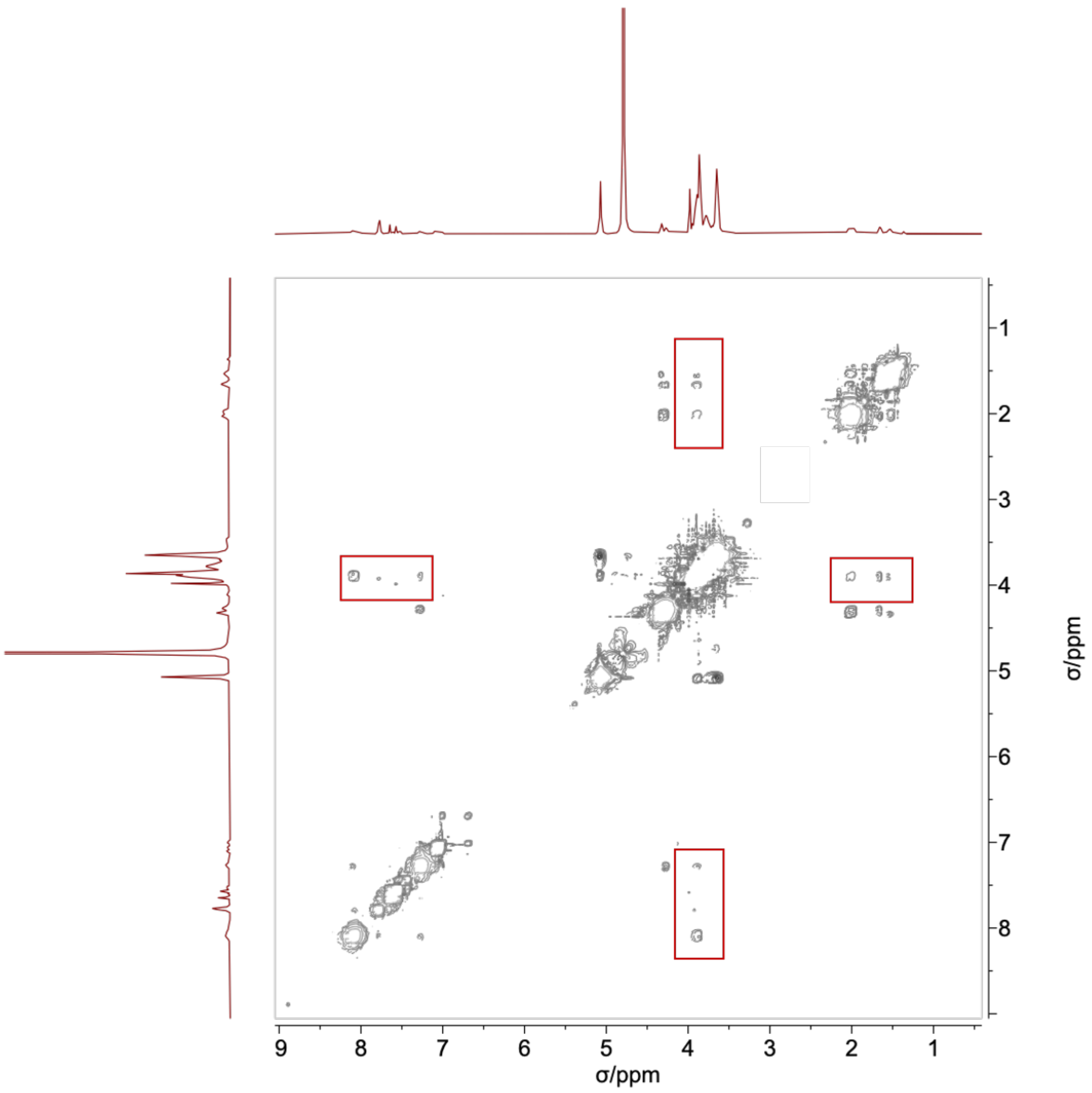

Supplementary Fig. 8. ^{1}H NMR 2D ROESY spectrum (400 MHz, D_2O, 298 K) of AzoC6/αCD (1:2 ratio). The red squares indicate the correlation peaks.

.

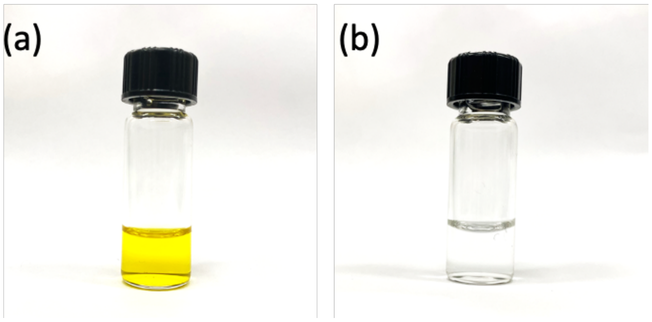

Supplementary Fig. 9. Digital images of (a) 0.05 M AzoC6 and (b) 0.1 M αCD aqueous solutions.

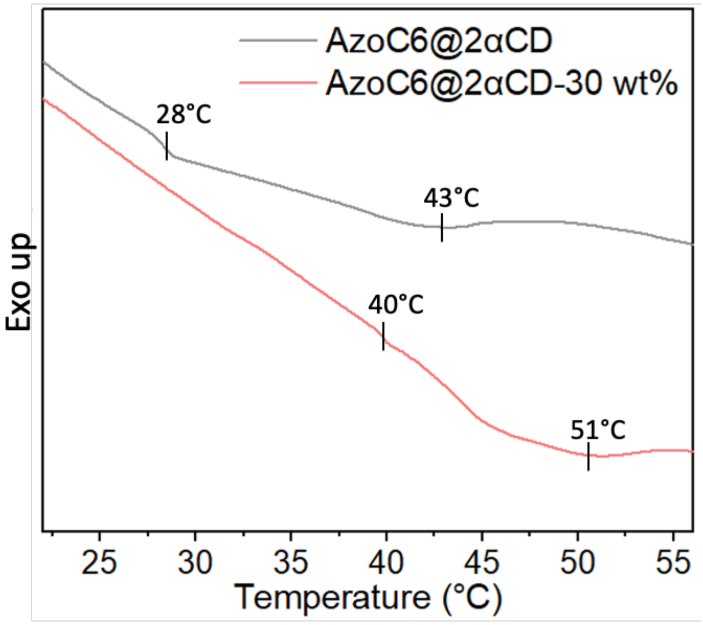

Supplementary Fig. 10. DSC heating traces of the AzoC6@2αCD hydrogel and the 30 wt% MXenegel. The glass transition and disassembly temperatures are indicated.

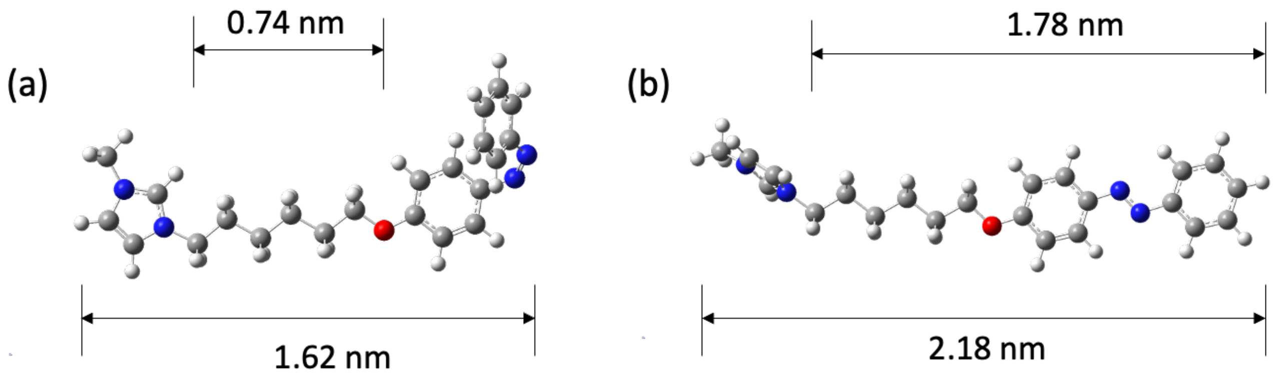

Supplementary Fig. 11. B3LYP/6-31G (d, p)-optimized structure of (a) cis-AzoC6 and (b) trans-AzoC6. The estimated lengths of different atoms are indicated.

To elucidate the host/guest ratio of the inclusions, the optimized geometry of the cis- and trans- AzoC6 is calculated by Gaussian using the B3LYP/6-31G (d, p) method. As shown in Supplementary Fig. 11b, the length of the trans-AzoC6 is ca. 2.18 nm, in which the hydrophobic part (alkyl chain and trans-azobenzene) is ca. 1.78 nm. The length of two and three αCD molecules is ca. 1.58 and 2.37 nm, respectively, indicating that one trans-AzoC6 guest can be included in a maximum of two αCD hosts. The cis-form AzoC6 molecule shown in Supplementary Fig. 11a, however, only has ca. 0.74 nm of alky chain that can be included in one αCD molecule because the size and polarity of the cis-azobenzene are not compatible with αCD cavity. Based on the scattering and simulating results, we deduce that the disassembly of the bilayer structure is triggered by the weakening of the hydrogen bonding between the AzoC6@αCD inclusions, which cannot stack into a lamellae structure, compared with the AzoC6@2αCD.

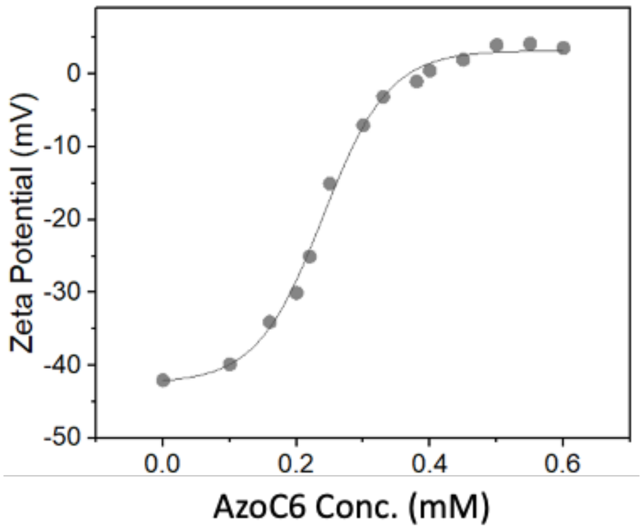

Supplementary Fig. 12. Zeta potential of MXene (0.5 mg/mL) at different concentrations of AzoC6 aqueous solution.

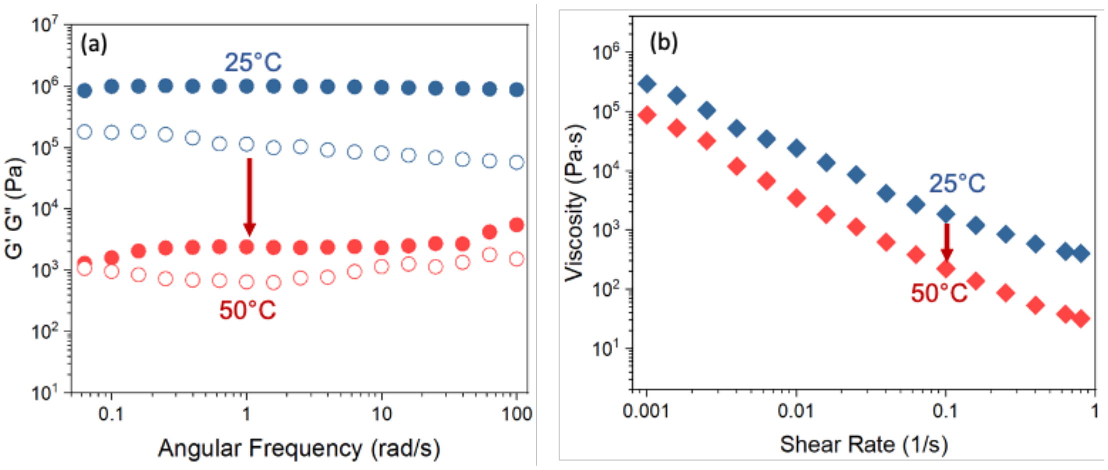

Supplementary Fig. 13. (a) Frequency sweep and (b) viscosity shear rate data of the 30 wt% MXenegel at different temperatures. The red arrows indicate the same sample at different temperatures.

The temperature ramping experiments are conducted in an 8 mm stainless steel plate geometry equipped with a water-circulating temperature-controlled system. The results shown in Supplementary Fig. 13 exhibit the changes of the storage modulus G' and loss modulus G'' and viscosity as a function of frequency oscillating frequency and shear rate, respectively. The G' and G'' of the 30 wt% MXenegel have dropped by nearly 3 orders of magnitude when the temperature increases from 25 to 50 °C. Similar viscosity change can also be observed in the operating shearing range. This is because the self-assembled nanostructure of the AzoC6@2αCD is mainly driven by the hydrogen bonds between the neighboring αCDs and the hydrophilic heads of the AzoC6 molecules. When the temperature increases above 50 °C, the nanostructure disassembles. The results obtained from the rheological measurements are in good agreement with the DSC results, in which the disassembly temperature at 51 °C is observed.

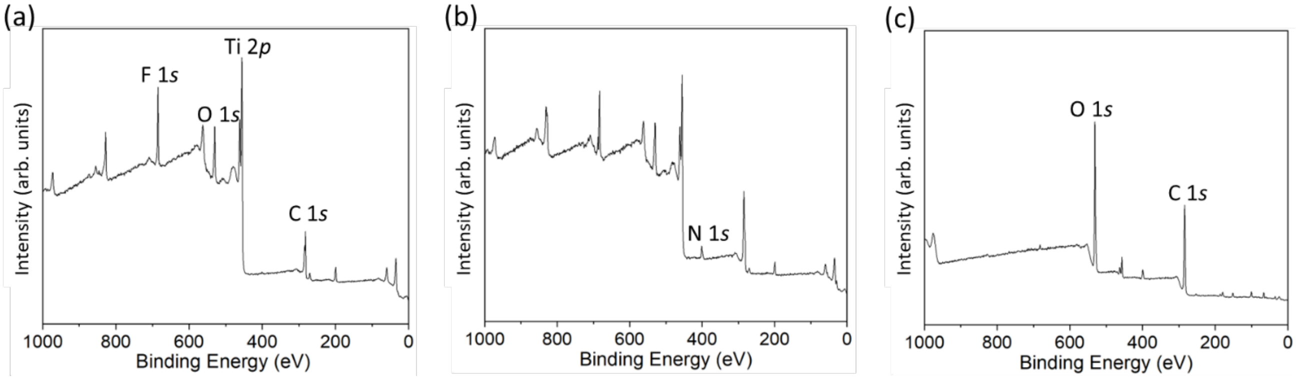

Supplementary Fig. 14. XPS survey spectra of (a) MXene, (b) MXene/AzoC6, and (c) MXenegel.

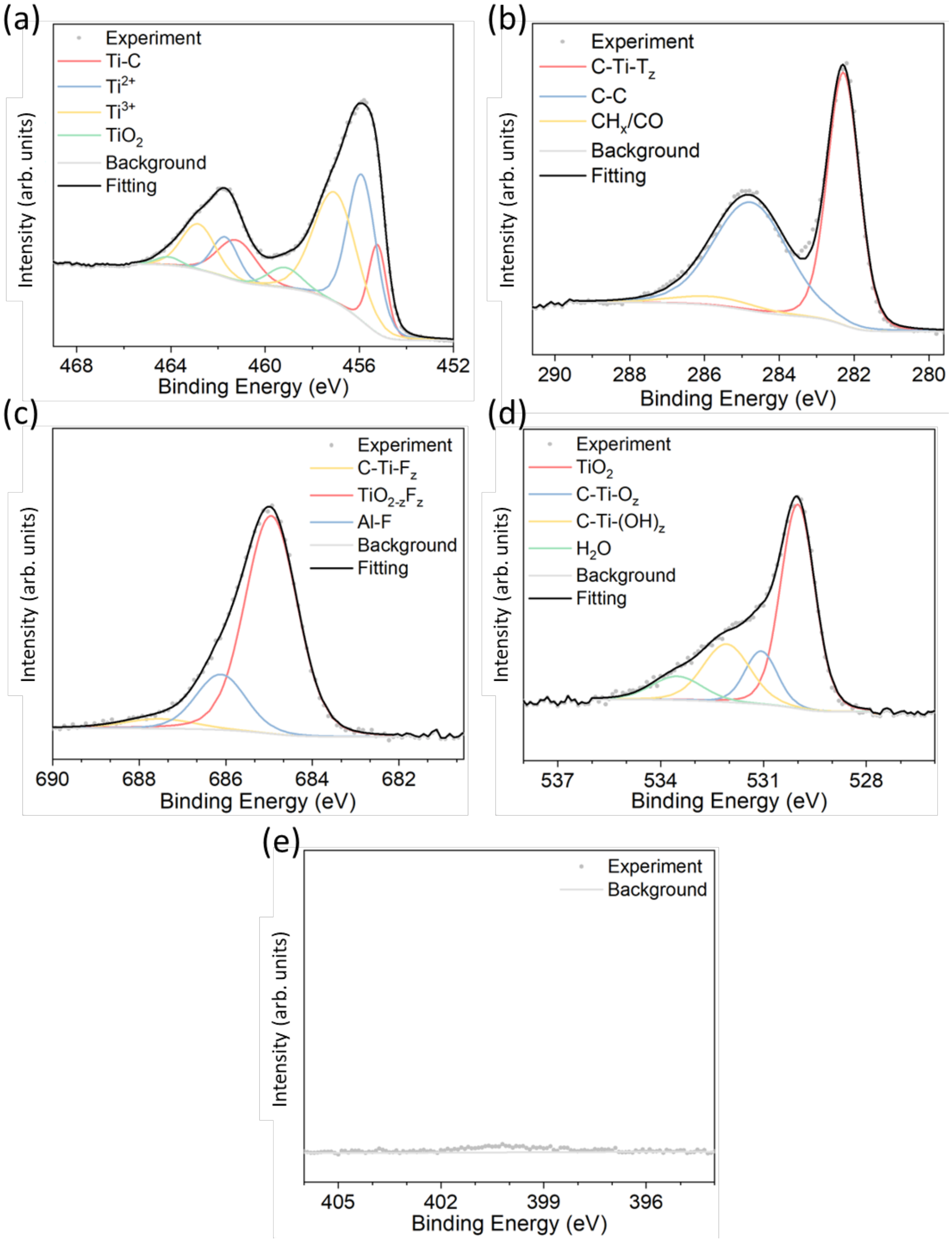

Supplementary Fig. 15. XPS component peak fits of MXene: (a) Ti 2*p*, (b) C 1*s*, (c) F 1*s*, (d) O 1*s*, and (e) N 1*s*. The sample is prepared in the aqueous state, filtered by a 200 nm filter paper, and washed with DI water several times.

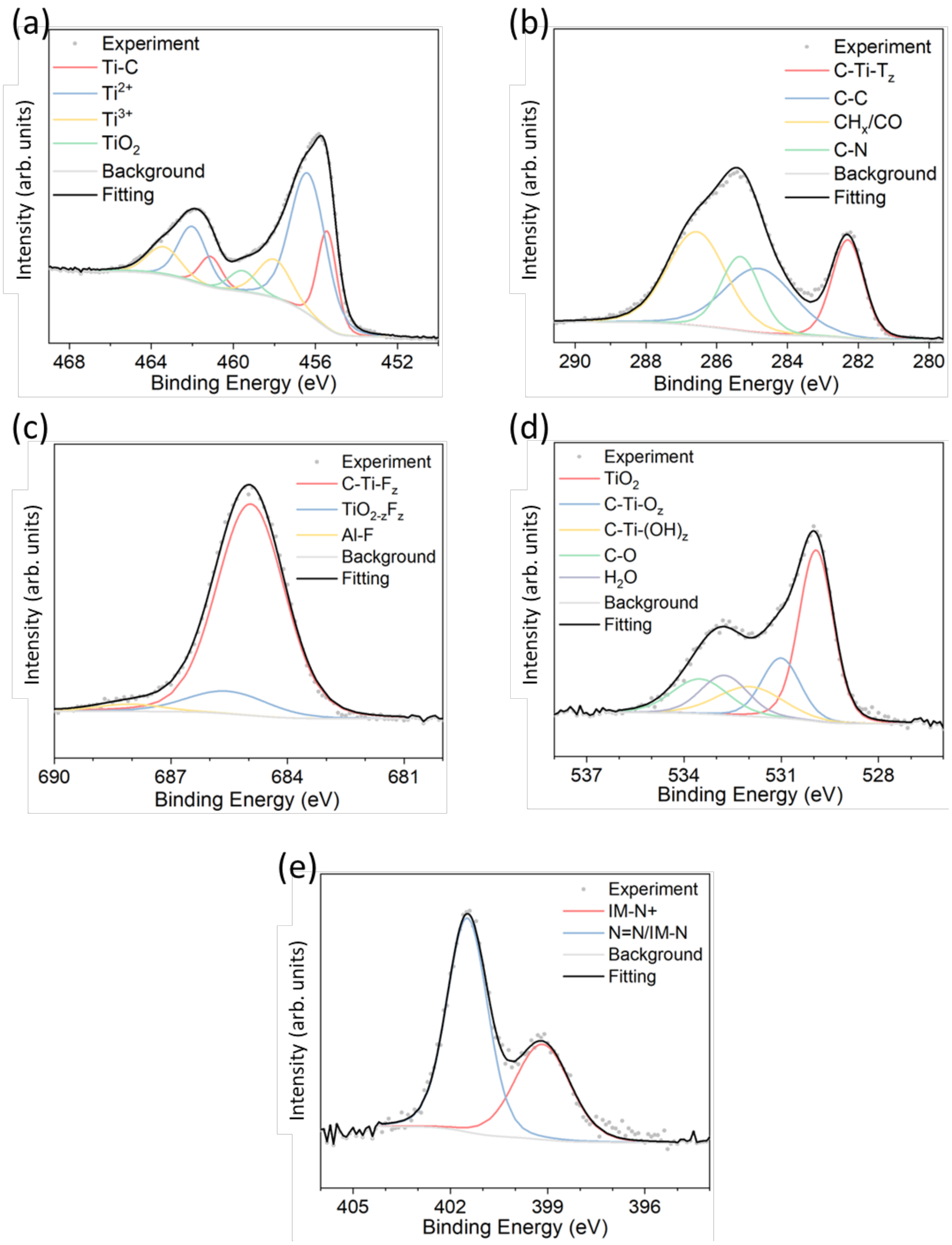

Supplementary Fig. 16. XPS component peak fits of MXene/AzoC6: (a) Ti 2p, (b) C 1s, (c) F 1s, (d) O 1s, and (e) N 1s. The sample is prepared in the aqueous state, filtered by a 200 nm filter paper, and washed with DI water several times to remove the unattached AzoC6 molecules.

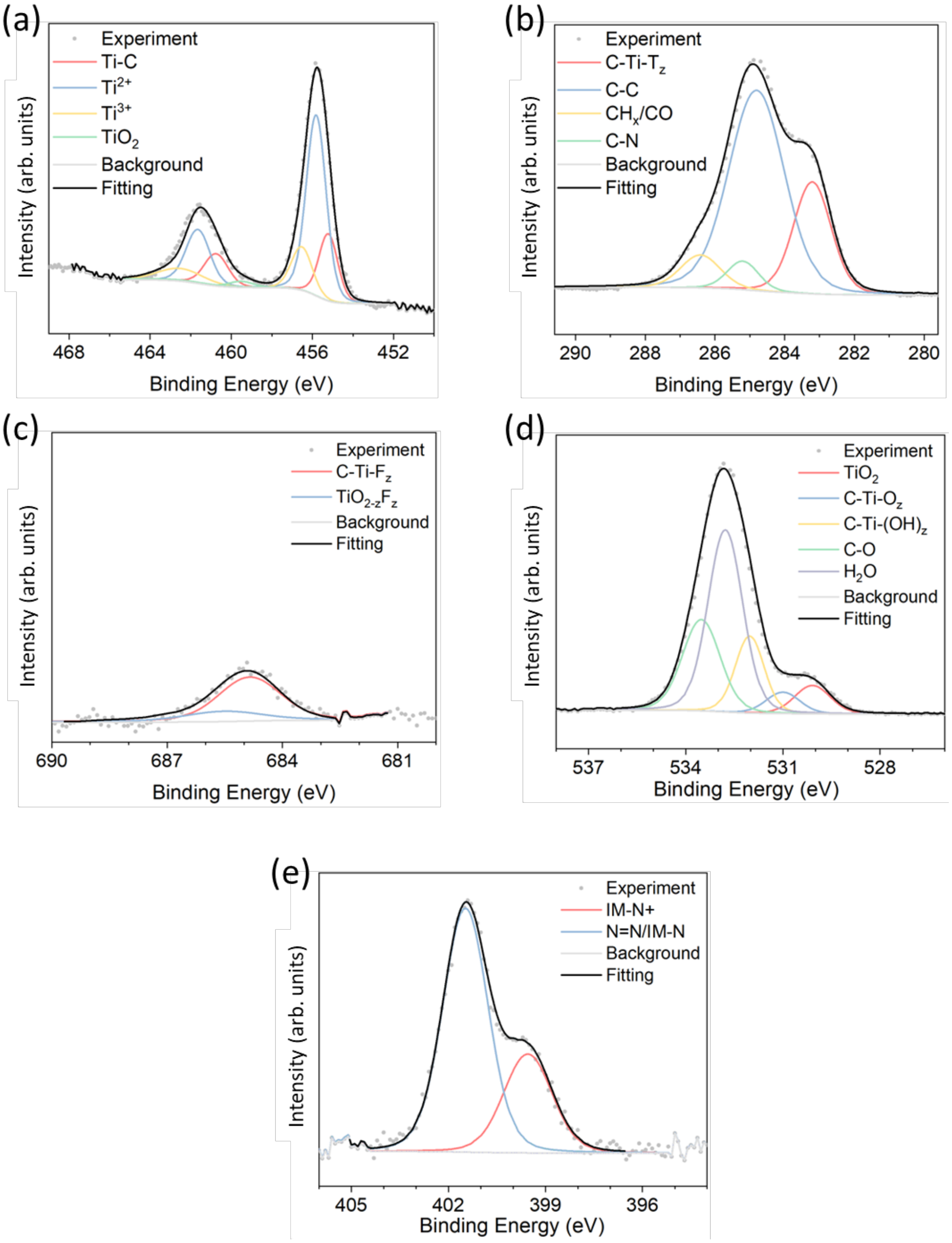

Supplementary Fig. 17. XPS component peak fits of the vacuum-dried MXenegel: (a) Ti 2*p*, (b) C 1*s*, (c) F 1*s*, (d) O 1*s*, and (e) N 1*s*.

Supplementary Table. 1. MXene ($Ti_3C_2T_x$) XPS peak fitting results

**Element**	**Element AT%**	**Binding energy (eV)**	**Component**	**Component AT%**	**FWHM**
**Ti 2*p*3/2 (2*p*1/2)**	21.1	455.2 (461.2)	Ti-C	20.5	0.9 (1.9)
		455.9 (461.7)	Ti^{2+}	33.6	1.4 (1.3)
		457.0 (462.8)	Ti^{3+}	40.1	2.1 (1.7)
		459.2 (464.1)	TiO_2	5.8	1.7 (1.3)
**C 1*s***	49.5	282.3	C-Ti-T_x	48.0	1.0
		284.8	C-C	47.6	2.3
		285.7	CHx/CO	4.4	2.6
**O 1*s***	20.3	530.0	TiO_2	55.3	1.2
		531.1	C-Ti-O_x	13.6	1.1
		532.1	C-Ti-(OH)x	21.6	1.6
		533.5	H_2O	9.5	1.7
**F 1*s***	9.1	687.5	C-Ti-F_x	4.6	1.9
		685.0	TiO_2-xF_x	76.8%	1.4
		686.1	Al-F	18.6%	1.4

Supplementary Table. 2. MXene/AzoC6 XPS peak fitting results

Element	Element AT%	Binding energy (eV)	Component	Component AT%	FWHM
**Ti 2*p*$_{3/2}$ (2*p*$_{1/2}$)**	21.2	455.4 (461.1)	Ti-C	20.8	1.1 (1.3)
		456.4 (462.0)	Ti^{2+}	54.0	2.0 (1.7)
		458.0 (463.4)	Ti^{3+}	18.4	1.9 (1.9)
		459.6 (464.0)	TiO_2	6.8	1.6 (4.0)
**C 1*s***	51.9	282.3	C-Ti-T_x	19.6	1.1
		284.8	C-C	28.2	2.3
		286.6	CH_x/CO	34.0	1.9
		285.3	C-N	18.2	1.3
**O 1*s***	13.0	529.9	TiO_2	42.2	1.3
		531.0	C-Ti-O_x	16.5	1.4
		532.0	C-Ti-$(OH)_x$	13.9	2.4
		533.5	C-O	14.0	2.0
		532.8	H_2O	13.4	1.7
**F 1*s***	8.5	685.0	C-Ti-F_x	86.2	2.1
		685.6	$TiO_{2-x}F_x$	11.0	2.5
		688.0	Al-F	2.9	2.0
**N 1*s***	5.4	399.2	N=N/IM-N	37.7	2.0
		401.5	IM-N^+	62.3	1.5

Supplementary Table. 3. MXenegel XPS peak fitting results

Element	Element AT%	Binding energy (eV)	Component	Component AT%	FWHM
**Ti 2*p*3/2 (2*p*1/2)**	2.0	455.2 (460.8)	Ti-C	23.3	1.1 (1.4)
		455.8 (461.6)	Ti^{2+}	55.7	1.2 (1.4)
		456.5 (462.5)	Ti^{3+}	17.0	1.2 (2.7)
		459.5 (463.3)	TiO_2	4.0	1.5 (4.3)
**C 1*s***	55.0	283.2	C-Ti-T_x	24.1	1.3
		284.8	C-C	63.4	1.9
		286.4	CH_x/CO	7.4	1.3
		285.2	C-N	5.1	1.1
**O 1*s***	37.6	530.1	TiO_2	7.3	1.3
		531.0	C-Ti-O_x	5.3	1.2
		532.0	C-Ti-$(OH)_x$	16.2	1.1
		533.5	C-O	25.1	1.4
		532.8	H_2O	46.1	1.3
**F 1*s***	0.7	684.9	C-Ti-F_x	77.6	1.9
		685.5	$TiO_{2-x}F_x$	22.4	3.3
**N 1*s***	4.7	399.5	N=N/IM-N	30.3	1.8
		401.5	IM-N^+	69.7	1.7

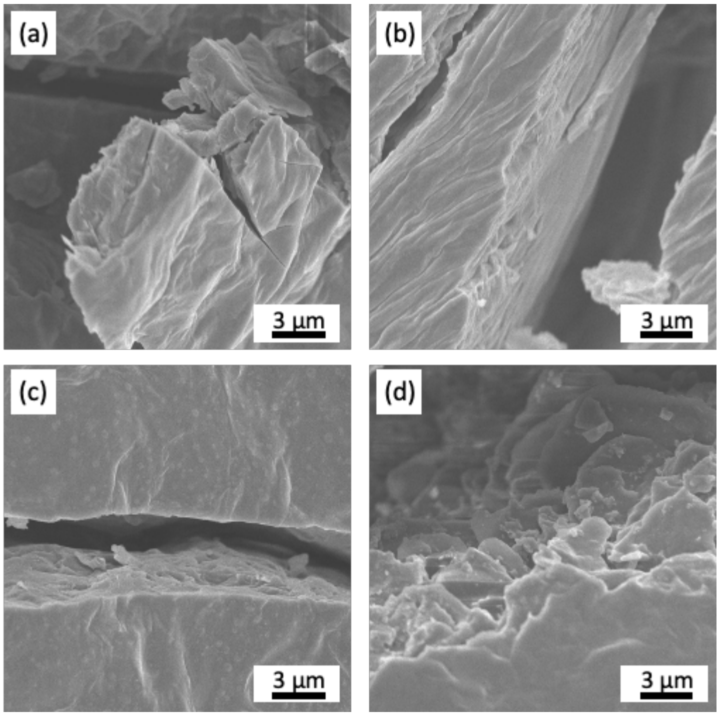

Supplementary Fig. 18. SEM images of vacuum-dried (a) 0.25, (b) 1.25, (c) 5, and (d) 30 wt% MXenegels.

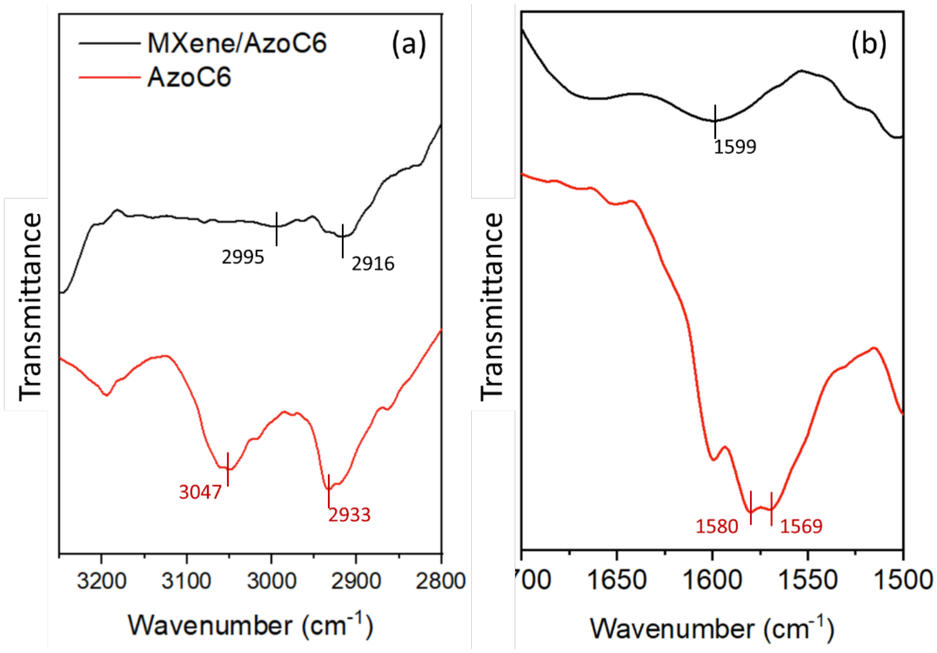

Supplementary Fig. 19. ATR-FTIR spectra of the AzoC6 powder and MXene/AzoC6 thin film: (a) C-H stretching and (b) imidazole ring stretching modes. The MXene/AzoC6 sample is rinsed with DI water several times to remove the unattached AzoC6 molecules. The characteristic peaks are also indicated.

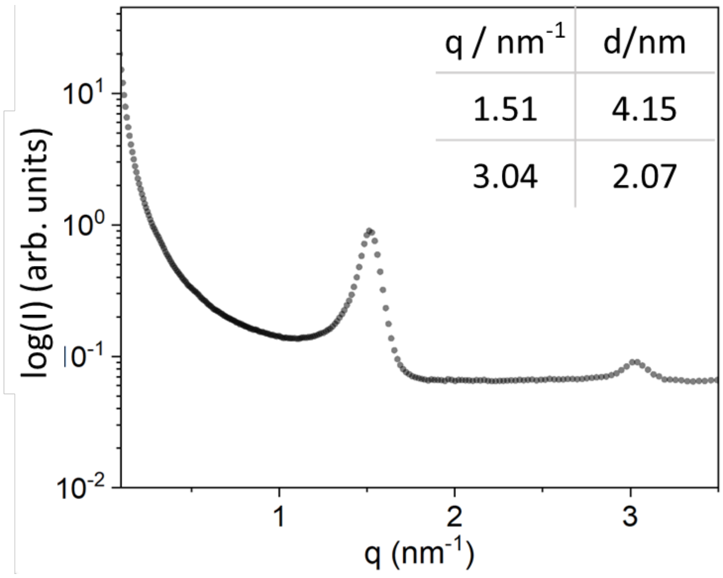

Supplementary Fig. 20. Integrated intensity profile of the vacuum-dried 30 wt% MXenegel. The q and the corresponding d-spacing values of the observed peaks are indicated.

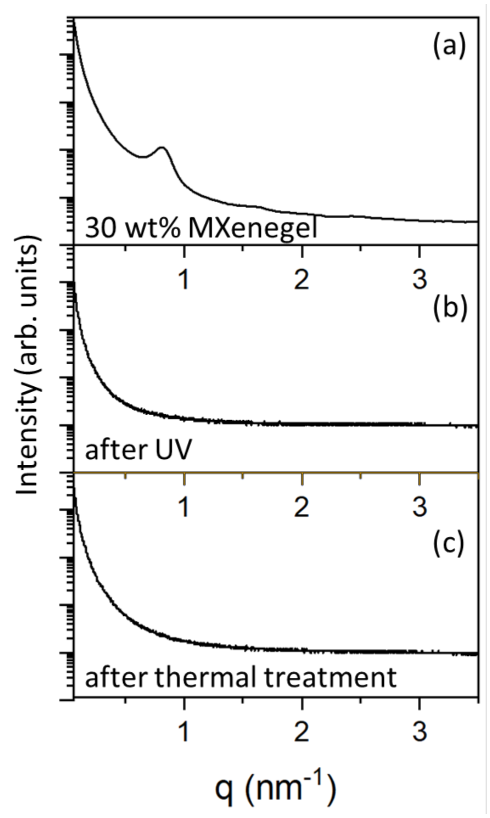

Supplementary Fig. 21. SAXS profiles of (a) the 30 wt% MXenegel and (b) after UV and (c) after thermal treatment (50 °C).

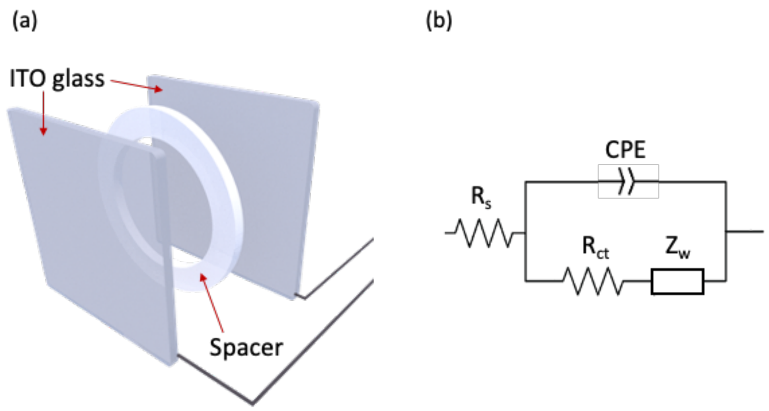

Supplementary Fig. 22. (a) Illustration of the electrode configuration for the electrochemical experiments. (b) The equivalent circuit applied in the fitting process.

Supplementary Fig. 22a shows the electrode configuration for the electrochemical measurements. A MXenegel is sandwiched between two ITO glasses, and a Teflon spacer is used as a spacer. In a 2-electrode setup, the voltage is applied between the working and the counter electrodes, and the current change is probed by an LCR meter. As displayed in Supplementary Fig. 22b, the equivalent circuit is used to fit the obtained raw data of the electrochemical impedance (EIS) spectra. The solution resistance R_s is in series with the parallel combination of a constant phase element (CPE) and an impedance of a faradaic reaction. The charge transfer resistance R_{ct} and the Warburg impedance Z_w are related to the electrochemical and diffusion processes' kinetics, respectively. Figure 5e shows the typical spectra obtained from the MXenegel, in which the Warburg line dominates the impedance plot for a reversible electrochemical system. The suppressed semicircle also implies that the system is dominated by mass transport because of the fast charge-transfer rate.

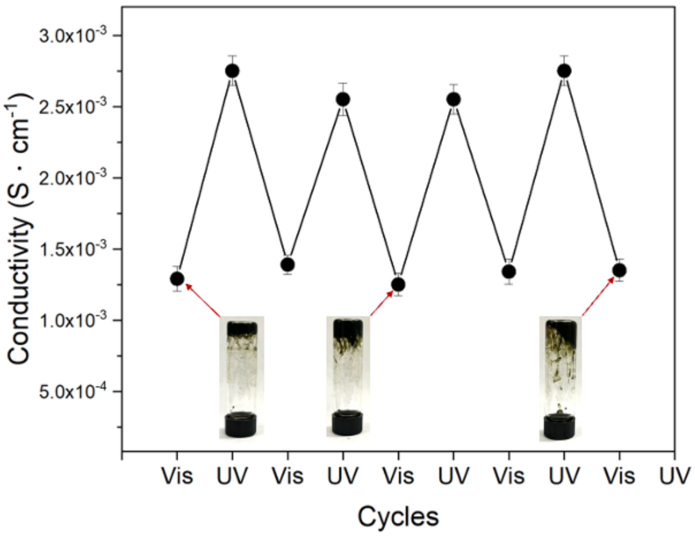

Supplementary Fig. 23. Conductivity analysis of the 30 wt% MXenegel under alternating UV and visible irradiations. The images of the MXenegel at the 0th, 2nd, and 4th cycles are shown. The error bars are the standard deviations calculated from 10 data points.

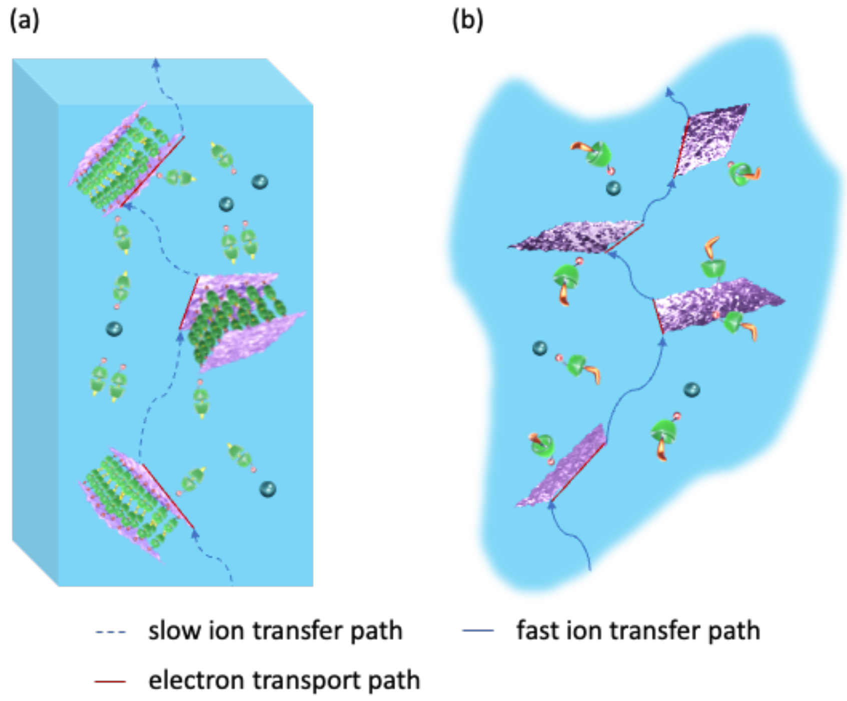

Supplementary Fig. 24. Proposed mechanisms of MXenegel conduction under (a) visible (gel state) and (b) UV (sol state) light irradiations. The conductivity of the MXenegel mainly depends on the concentration of the conducting dopants (MXene) and the physical condition of the matrix (AzoC6@2αCD). Conductions take place through the electron transport of the MXene flakes and the ion migrations.
